# The HIV and SARS-CoV-2 Parallel in Dentistry from the Perspectives of the Oral Health Care Team

**DOI:** 10.1177/2380084420961089

**Published:** 2020-09-18

**Authors:** M. Brondani, L. Donnelly

**Affiliations:** 1 Division of Dental Public Health, Faculty of Dentistry, University of British Columbia, Vancouver, BC, Canada; 2 Faculty of Dentistry, University of British Columbia, Vancouver, BC, Canada

**Keywords:** COVID-19, social unrest, stigma, qualitative study, interviews, Canada

## Abstract

**Objectives:**

The aim of this study was to unravel the professional and social consequences of COVID-19 as compared with the AIDS pandemic according to oral health care providers, staff, and administrators.

**Methods:**

An exploratory qualitative inquiry via at-a-distance, semistructured interviews engaged a purposefully recruited sample of oral health care team workers in British Columbia. Interviews took place between April 20 and May 15, 2020; they were audio recorded, transcribed verbatim, and deidentified for interactive thematic analysis. An inductive process of coding was used to identify themes, subthemes, and categories of information.

**Results:**

Forty-five interviews were conducted with 18 dentists, 12 dental hygienists, 6 certified dental assistants, and 9 administrators; 22 were females. Interviews each lasted an average of 48 min. After the transcripts were coded, 3 subthemes emerged: 1) personal protective equipment and universal precautions as commonsense approaches to care during both pandemics; 2) an (un)collapsed world in terms of global lockdowns; and 3) social unrest in terms of the potential for stigma and discrimination caused by both pandemics. These subthemes made up the COVID-19–AIDS parallel theme.

**Conclusion:**

This study explored the extent to which the current COVID-19 pandemic is leading to professional and social consequences when a parallel is drawn with the AIDS pandemic. This is the first qualitative study that identifies the potential social unrest of the pandemic from the perspective of oral health care providers and administrators. Future studies should include other providers across Canada, as well the patients receiving oral health care during this pandemic.

**Knowledge Transfer Statement:**

The COVID-19 pandemic has unraveled potential societal implications in a parallel to the HIV/AIDS era from the perspectives of oral health care providers and their staff. Such implications are changing the way that oral health care is delivered; it may also be leading to social unrest in the form of stigma and discrimination. This study discusses some of these implications from the perspective of oral health care providers and administrators.

## Introduction

Although virus outbreaks continue to emerge and threaten public health across the globe, the current novel coronavirus disease 2019 (COVID-19) pandemic caused by SARS-CoV-2 (severe acute respiratory syndrome coronavirus 2; Zhu et al. 2020) is perhaps the only infection in modern history to bring oral health care to a halt, probably because of its route of transmission via respiratory and saliva droplets (Proffitt 2020). COVID-19 also seems to be bringing sweeping changes to personal protective equipment (PPE), universal precautions (UPs; McCarthy 2000), and the way that oral health care is delivered via new clinical protocols and procedures that minimize or do not generate aerosols (Coulthard 2020). These changes can be compared with the modifications that HIV (human immunodeficiency virus)—implicated in the development of AIDS (acquired immunodeficiency syndrome)—brought to the practice of oral health care back in the 1980s (DePaola 2003). Although the current PPE and UPs were introduced in dentistry as a response to the HIV outbreak, the HIV/AIDS era brought to the surface a much more grim reality: the stigma and discrimination (Mawar et al. 2005) still faced by many individuals around the world and in Canada when accessing oral heath care (Donnelly et al. 2016; Brondani, Donnelly, and Postnikoff 2016; Brondani, Phillips, et al. 2016; Jessani et al. 2019). Similarly, stigma is currently faced by certain populations due to SARS-CoV-2 (Hanasoge et al. 2020; Logie and Turan 2020; Nature 2020). However, the extent to which COVID-19 has affected access to professional oral health care and led to societal implications from the perspectives of providers and administrators remains unknown. The main objective of this study was to unravel the potential professional and social consequences of COVID-19 according to oral health care providers, staff, and administrators in British Columbia, Canada. Of note, the study presented herein is part of a larger qualitative project entitled “Structural Preparedness during the COVID-19 Pandemic and the Provision of Urgent Oral Health Care.” Although that project was not designed from the outset to explore the COVID-19–AIDS parallel, the issue frequently emerged during interviews with members of the oral health care team and is reported accordingly.

## Methods

The University of British Columbia’s Behavioural Research Ethics Board approved this study (H20-01147). We employed a qualitative inquiry method via individual interviews with dentists, dental hygienists, certified dental assistants, and staff (e.g., administrators and front desk personnel) from across British Columbia between April 20 and May 15, 2020. Participants were purposefully informed about the study via an email distributed to a province-wide professional list and through snowball sampling via word of mouth. Inclusion criteria covered any of the target audience that was unemployed (e.g., offices or practices were closed) or continued to work to treat dental emergencies after the curtailment of oral health care services in British Columbia on March 16, 2020 (BC Dental Association 2020). Inclusion criteria targeted participants of any gender and with various years of experience; however, we attempted to establish a somewhat even representation for professional roles (e.g., dentists, dental hygienists), gender, and years of experience in that role (e.g., <10 y and >10 y) whenever possible. Participants contacted the first author, who then shared information about the study, the interview process, and the informed consent. The interviews where scheduled in the order in which the emails were received, and they were conducted at a distance via phone, Zoom video communication, or Microsoft Skype platform at a day and time convenient for the participant, given the recommendations for physical distancing during the pandemic. Interviews were conducted by one of the authors or by a hired research assistant; interviewers were calibrated by interviewing the first 2 participants in a group format to refine the interview guide. Interview questions included but were not limited to the following: 1) “What do you know about the COVID-19 outbreak?” 2) “What do you know about the transmission of the virus?” 3) “Why is this pandemic relevant to oral health care?” 4) “What do you understand by being prepared to provide oral health care during the pandemic?” While we did not plan to ask questions about HIV/AIDS in particular, the nature of qualitative inquiry allowed us to probe for that information after participants willingly compared the 2 pandemics from various perspectives. It is important to note that participants used “HIV” and “AIDS” interchangeably, even though HIV is the virus implicated in the development of AIDS and not all patients carrying HIV develop AIDS.

**Figure 1.**
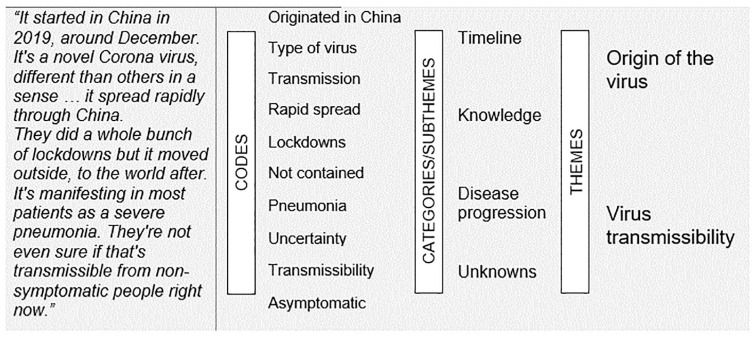
Thematic analysis via the coding process of a transcript excerpt focused on knowledge about COVID-19.

A total of 18 dentists (7 females), 12 dental hygienists (11 females), 6 certified dental assistants (all females), and 9 administrators (5 females) participated, ensuring saturation of the information with 45 interviews. Interviews lasted an average of 48 min, led to 51 h of audio recordings, and generated >650 pages of transcripts. Participants received a $150 honorarium in appreciation for their time. Demographic data on gender, profession, work location, and years of practice were documented for information only. Audio recordings were transcribed verbatim, deidentified in the order in which they were completed, and analyzed interactively; transcripts were explored for codes and themes concomitantly with the interviews (Smith 2005). Between April 29 and May 21, 2020, the 2 authors independently analyzed 22 transcripts each after 1 round of interactive coding of the first transcript to increase rigor of the audit trial. As the authors independently coded and conducted the thematic analyses of different transcripts, they met constantly via conference calls to discuss the codes, categories, and themes identified for consensus. Figure 1 presents an example of this coding process in 1 excerpt from 1 of the transcripts, as we have done previously (Brondani et al. 2007; Donnelly et al. 2016; Feng et al. 2018). As shown in the figure, “coding” refers to an inductive process of identifying specific ideas or labels in the form of a word or words (e.g., “type of virus,” “originated in China,” “lockdowns”) within sentences or excerpts from the transcripts. Similar and related codes are then grouped to represent a specific category or subtheme (e.g., “knowledge”). Related categories or subthemes are clustered to represent a main theme, including “origin of the virus.” This study opted for an inductive coding process grounded on the qualitative data, rather than a deductive process based on a predefined set of codes, so that important ideas were not overlooked (Smith 2005).

## Results

**Figure 2.**
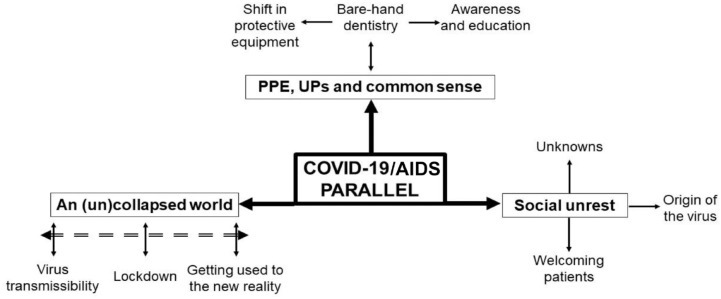
Thematic visualization of the COVID-19–HIV/AIDS parallel. PPE, personal protective equipment; UP, universal precaution.

From the 45 interviews, 33 participants discussed HIV/AIDS in one way or another, which allowed us to identify 3 subthemes: 1) PPE, UPs, and common sense; 2) an (un)collapsed world; and 3) social unrest. These 3 subthemes were identified under the COVID-19–AIDS parallel as the main theme and are presented in Figure 2. To contextualize the themes, the professional role, work location, and years of experience are added to the excerpts from the transcripts when applicable.

### Theme 1: PPE, UPs, and Common Sense

Across all the interviews, there was no question that the current practice of dentistry and dental hygiene could not be envisioned without the use of protective gloves and masks as a minimum: “We take the necessary precautions to promote a safe environment for us and our patients by removing gloves and masks after every appointment” (dental hygienist with 15 y of practice in the interior of British Columbia). Specifically, 21 participants took the opportunity during their interview to draw parallels between HIV and SARS-CoV-2 in terms of PPE and UPs. For a certified dental assistant working for >30 y in the same office in northern British Columbia, barehand dentistry was the norm until the arrival of HIV:

When I first started we didn’t use masks or gloves. But that all changed—we were all tested for HIV. And we were very mindful of that and that was when we started wearing glasses, and masks and gloves. Now you would never consider working on somebody without any of it.

The idea of pre–HIV era barehand dentistry was highlighted by 9 other participants. For 7 other participants, including a dentist who was practicing in Vancouver Island in the 1980s, the parallel between COVID-19 and AIDS surfaced when commenting on the sterilization of instruments:

I think it’s just like when HIV/AIDS happened in the 80s. We used to disinfect our hand instruments with CaviCide, and there was not a big deal. Then [autoclaves] became mandatory. Now [with COVID-19] I think it’s going to definitely be changes in a lot of the sterilization again.

In the words of a dentist who worked on Vancouver Island for 4 decades, “I remember in the 80s we used to wash any linen, apron, or towel when we were concerned about HIV because we didn’t have enough knowledge. We washed them in the washing machine under extremely hot water.”

However, for many participants, unrest around COVID-19 was related to the way that the virus can be transmitted during an appointment: “If before we were worried about HIV and blood, now we will be much more concerned with aerosols and droplets, and they are everywhere, every time care is provided” (certified dental assistant working in Vancouver for >20 y). This unrest seems to be predominantly influenced by a lack of a full understanding about transmissibility and the perceived impact of the virus on the cost and time associated with minimizing or eliminating the risk of transmission within a dental setting.

### Theme 2: An (Un)Collapsed World

All interviewees acknowledged that COVID-19 has affected their lives in one way or another. According to a senior administrator working for almost 30 y at a community dental clinic in the greater Vancouver, “this COVID-19 pandemic is impacting both the demand and supply sides of dental care, and has also major implications in the lives of our patients who might have lost their jobs, and locked at home, and are now struggling to feed their families.” In fact, the lockdown experienced by many cities and countries worldwide due to the COVID-19 pandemic was questioned by a dental hygienist who started working in the 1980s in the greater Vancouver area, as she recalled what happened when HIV/AIDS emerged:

HIV has infected millions of people and if you remember back then, nothing shut down because of AIDS. The whole world didn’t shut down, right. I mean, it was definitely awful, but it wasn’t like the whole world pandemic that we now have with COVID-19.

While considering the lockdown, 12 participants, including a junior dentist <6 y into practice in Northern British Columbia, brought up the issue of travel bans: “It seems intuitive to close the border if you want to prevent the spread of a disease and contain the infection. But at what cost? I’m not sure it is even working the way it should.” A similar yet unparalleled reality was drawn among the SARS outbreak in the early 21st century, HIV, and COVID-19 according to a senior dental hygienist:

For one, Canada did not do well with SARS because there was just not enough understanding, there wasn’t enough response. With HIV, we just treated everybody as infected. But now there is a huge earthquake in our lives that this [COVID-19] is causing.

One of the participating dentists who started practicing in a small city in British Columbia in the earlier HIV pandemic mentioned the issue of transmission of the virus when drawing an (un)parallel between COVID-19 and HIV:

I do not think they are comparable, no. What comes to mind is that HIV is [a] blood- and other body fluids–borne disease. It is almost impossible to get in the dental office compared to COVID-19 through saliva droplets, which is everywhere.

For 17 other participants, the relative unrest around COVID-19 will likely be transient as it was for HIV, and some of the changes might compose a new norm for years to come.

### Theme 3: Social Unrest

The comparison between COVID-19 and HIV also surfaced in terms of profiling and stereotyping those who might have the disease, as mentioned by 6 participants. The following dialogue between the interviewer and a junior practicing dentist in the interior of British Columbia for 8 y exemplifies this:

D.M.D.: I think this disease [COVID-19] . . . reminds me of the HIV epidemic.

Interviewer: What do you mean?

D.M.D.: I heard a little bit about the reaction against the Chinese community because people assume that if you are Chinese, you have the disease, or that you caused [it]. So, the same with HIV and being a gay man in particular at the time, as they were blamed for [AIDS].

For numerous participants, the professional duty remains to make all patients comfortable in the dental chair regardless of who they are or what they have. For a dental hygienist with >35 y of experience working in the Vancouver area, it was a matter of welcoming all patients: “With AIDS I felt confident and I wanted to welcome all patients. . . . I didn’t want them to feel stigmatized, and the same with COVID patients now.” We were also told, “We just treat everybody the same way. [It] is that simple, and it should also be the same now” (dental hygienist working on Vancouver Island since 1985).

The protocol of doubling gloves and masks during the HIV outbreak might come back during the COVID-19 pandemic according to a senior dentist working for >30 y in the interior of British Columbia. He told us that COVID-19 “is going to bring back that piece of protocol [with] the double gloving during AIDS.” Another (un)parallel was related to the way that the viruses implicated in the development of those diseases are transmitted. We were told by a dentist with 38 y of experience in Vancouver that

HIV, it is of low [transmission], if any, in a dental office, with our current universal precautions. As a blood-borne disease, it is much more difficult to get in the dental office, for example, compared to this one [COVID-19] that is much easier via saliva droplets and aerosols.

According to a young practicing dentist, the almost identical parallel between COVID-19 and HIV/AIDS was based allegedly on the origin of the viruses:

This information is coming from everywhere. What I know is that [COVID-19] is about a virus, allegedly from a live animal meat market in Wuhan, China. Isn’t that the same history behind HIV back in the 80s, from slaughtering monkeys in the [African] jungles, that was transmitted to us?

## Discussion

Our main objective with this qualitative inquiry was to unravel the potential professional and social implications of the COVID-19 pandemic according to members of oral health care teams from across British Columbia, Canada. Figure 2 shows the intertwined relationship of the COVID-19–AIDS parallel with 3 subthemes identified from 33 transcripts: 1) PPE, UPs, and common sense (from barehand dentistry to education and awareness); 2) an (un)collapsed world (from lockdowns to a new reality); and 3) social unrest (from unknown to welcoming patients). These 3 subthemes might overlap in meaning and relevance and are mapped in relation to the main theme indicated via single solid arrows. Subtheme are composed of their respective codes. While some of these codes are linked with the subtheme via solid double arrows to denote a mutable relationship (e.g., an [un]collapsed world and “lockdown”), others are linked within themselves to portray interdependency (e.g., barehand dentistry with “awareness and education” and “shift in protective equipment”). The dashed double arrow shows a reversible relationship between codes, such as “virus transmissibility” and “lockdown”: as new information and a better understanding about the transmission of the virus emerge, some lockdowns may be eased.

As acknowledged by many participants, the arrival of HIV at a time when dentistry was practiced mostly barehanded introduced the concept of PPE and UPs (McCarthy 2000) as providers became more aware and educated about infection control (DePaola 2003; McCarthy 2000). PPE and UPs are now part of a commonsense approach to infection control that treats every patient and one’s bodily fluids as infectious for any blood-borne pathogen, even when the patient is unaware of the infection or is asymptomatic. HIV transmissibility is now widely known, and the development of AIDS has been successfully curtailed by medications (Hulla and Montaner 2013), which is not yet the case for COVID-19 as of July 2020 (Rabby 2020; Sanders et al. 2020). Participants also discussed the fact that awareness and education around HIV/AIDS helped them to better understand the risks of transmission of that disease in the context of a dental office. The same can be said about COVID-19, as new information about disease transmission can only increase professional knowledge and improve practice skills in providing care to patients who are SARS-CoV-2 positive (Li et al. 2020).

Unlike COVID-19, the AIDS era did not cause a global collapse and lockdowns, as the diseases have very different transmissibility pathways, via respiratory droplets (Zhu et al. 2020) and bodily fluids (Shaw and Hunder 2012), respectively. Although transmission seems to be heightened by a potential airborne transmission route relevant for the spread of SARS-CoV-2 (Setti et al. 2020), travel bans and restrictions have been widely criticized (Petersen et al. 2020), given that they failed to effectively affect the epidemic’s trajectory (Chinazzi et al. 2020) when used at random without careful risk assessment (Devi 2020). However, unlike the travel bans for individuals who are COVID-19 positive that are being lifted <4 mo after the initial outbreak, >40 countries still impose travel restrictions on those living with HIV almost 4 decades after the first cases were reported in North America (UNAIDS 2019). In fact, it has been only 10 y since the United States lifted a 22-y restriction on immigration and travel for those living with HIV/AIDS (Winston and Beckwith 2011). These few months of social/physical distancing, self-isolation, and travel restrictions due to COVID-19 have already decreased the workforce across all economic sectors, have caused job loss levels not seen since the 1930s Great Depression, and have created economic crises and recessions globally (Nicola et al. 2020).

Although physical distancing interventions seemed to reduce the COVID-19 incidence across many countries (Islam et al. 2020), there is a harsh social reality emerging. As voiced by at least 6 participants, the COVID-19 pandemic is leading to a number of hate crimes and to anti-Asian sentiment, as China is being blamed for the outbreak (Chen and Trinh 2020; Schild et al. 2020). The way that the COVID-19 pandemic is fueling racism and discrimination against Asian individuals is unsettlingly, similar to the constant prejudice that gay men still face in association with HIV/AIDS (Mawar et al. 2005), particularly when accessing oral health care (Brondani, Donnelly, and Postnikoff 2016; Donnelly et al. 2016; Jessani et al. 2019). Such stigma, discrimination, and prejudice are a constant for certain ethnicities and religious groups that have been experiencing xenophobia (Suleman et al. 2018; Hanasoge et al. 2020) and anti-Semitism (Kogan 2017). As advocated by Chen and Trinh (2020), “if we can unite to overcome a pandemic of epic proportions, certainly we can also confront the socioracial issues [from it].”

The idea brought forward by 1 participant to double glove when treating patients with COVID-19 does not ease the experience of stigma, and it is probably unjustified given the transmission route of SARS-CoV-2. Despite double-gloving techniques being suggested as an effective means of infection control for high-risk medical surgical procedures involving patients who may have blood-borne infections (Padhye et al. 2011; Lipson et al. 2018), some patients who are HIV positive may feel stigmatized if they see the dentist overusing this measure (Patel et al. 2015). Additionally, in terms of the origin of both viruses, participants made reference to the fact that SARS-CoV-2 and HIV are zoonotic in nature (Sharp and Hahn 2011), albeit from different animals and a consequence of cross-species infections. However, the extent to which the parallels regarding the origin of the 2 diseases are implicated in the current social unrest discussed by our participants remains unknown.

As voiced by the participants of this study, the COVID-19 pandemic has unraveled potential societal implications in a parallel to HIV/AIDS. Such implications are likely changing the way that oral health care is delivered for the time being, and it may be leading to social unrest in the form of stigma and discrimination.

Saturation helped to achieve rigor in this qualitative study, as no new information was provided on the issue under investigation after 45 interviews (Saunders et al. 2018). Rigor was also attained by conducting member checking at different stages of the study, where participants were given the opportunity to read their transcripts and/or the thematic analysis and/or the final report (Birt et al. 2016); however, only 2 participants provided member checking on the thematic analysis, and they did not suggest any changes.

Our study has several limitations. The focus on British Columbia may prevent generalization despite the data reaching saturation. This qualitative research was also highly contextual, as it took place within western Canada in a specific period during a worldwide pandemic and might not be readily duplicated. The remote mode of the interviews might have been impersonal when compared with the commonly employed face-to-face method and might have prevented us from assessing essential nonverbal cues; yet, it gave us the opportunity to engage with participants from across the province without the need to travel. The honorarium paid to the participants may have attracted those who might have expressed their ideas in a more socially desirable manner. However, the interviewers let the participants be at ease and openly discuss their thoughts. Although we tallied the number of times that HIV-related information, codes, and subthemes were assigned within the transcripts, we did not perform any statistical evaluation of these frequencies, nor did we interpret such frequency as a sign of relevance or importance necessarily. Future studies are necessary to include the opinions of a larger group of oral health care providers in British Columbia and across Canada, given the interprovincial differences in professional regulations and population composition. The perspectives of the patients themselves should be sought, particularly around the social unrest experienced as recipients of oral health care during this pandemic. Last, follow-up inquiries should explore the roots of COVID-19 stigma, how prior destigmatizing interventions can contribute to tackle the discrimination related to SARS-CoV-19, and how different stigmatizing conditions influence one another.

## Conclusion

This study explored the extent to which the current COVID-19 pandemic is leading to consequences, socially and in terms of professional practice, when a parallel is drawn with the AIDS pandemic, according to the views of oral health care providers and administrators. The interactive thematic analysis revealed the major theme as the COVID-19–AIDS parallel with 3 subthemes: common sense around PPE and UPs, a world that is collapsed in some ways but not others, and a potential social unrest surfacing in terms stereotyping certain patients. This is the first qualitative study that identifies the potential implications of the pandemic when compared with the HIV/AIDS era to the practice of oral health care. Future studies should include oral health care providers across Canada, as well as the patients receiving oral health care during this pandemic.

## Author Contributions

M. Brondani, contributed to conception, design, data acquisition, analysis, and interpretation, drafted and critically revised the manuscript; L. Donnelly, contributed to data analysis and interpretation, critically revised the manuscript. Both authors gave final approval and agree to be accountable for all aspects of the work.
